# Fracture of the lesser trochanter as a sign of undiagnosed tumor disease in adults

**DOI:** 10.1186/s40001-015-0167-8

**Published:** 2015-09-04

**Authors:** Christian Herren, Christian D. Weber, Miguel Pishnamaz, Thomas Dienstknecht, Philipp Kobbe, Frank Hildebrand, Hans-Christoph Pape

**Affiliations:** Department of Trauma and Reconstructive Surgery, University Clinic RWTH Aachen, Pauwelsstraße 30, 52074 Aachen, Germany

**Keywords:** Fracture, Lesser trochanter, Metastatic, Tumor disease

## Abstract

Isolated avulsion fractures of the pelvic ring are rare and occur predominantly in adolescent athletes. Isolated fractures of the lesser trochanter are reported to be pathognomic for tumor diseases in adults. We present a case of a female patient with an isolated avulsion of the lesser trochanter after treatment by her chiropractor. After staging examination, we determine the diagnosis of a left-sided carcinoma of the mamma. Additional imaging shows multiple metastases in liver, spine and pelvis. Palliative therapy has started over the course of time. We suggest, on suspicion of a malignant metastatic process, further investigation.

## Background

Isolated fractures of the lesser trochanter are uncommon and have been reported predominantly in adolescent athletes [1]. This injury is caused by severe impact, usually in context of contact sports and following a forceful and sudden muscle contraction of the iliopsoas with avulsion fracture of the apophysis. In adults, similar mechanism may result in muscle sprains [2]. Thus, isolated fractures of the lesser trochanter are a rare presentation of hip fractures in adults. In the literature, a few case reports describe this type of fracture as a sign of metastatic tumor diseases [3]. We present a case of a female patient with an isolated fracture of the lesser trochanter revealing the first manifestation of metastatic breast cancer.

## Case presentation

**Figs. 1, 2:**
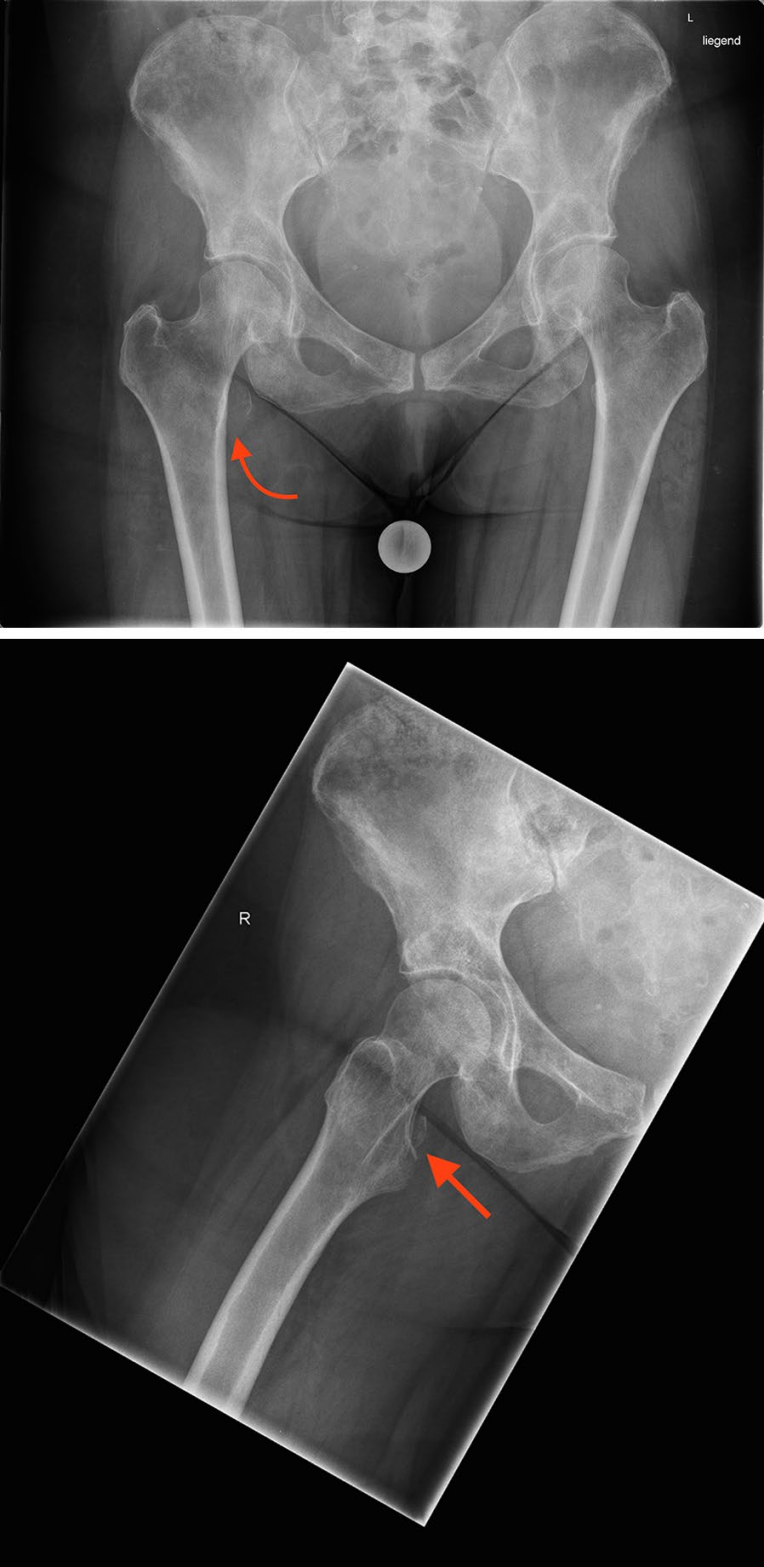
X-ray of the pelvis in AP view shows an isolated lesser trochanteric fracture (see *red arrows*) beside multiple osteolytic processes of the pelvis

**Fig. 3:**
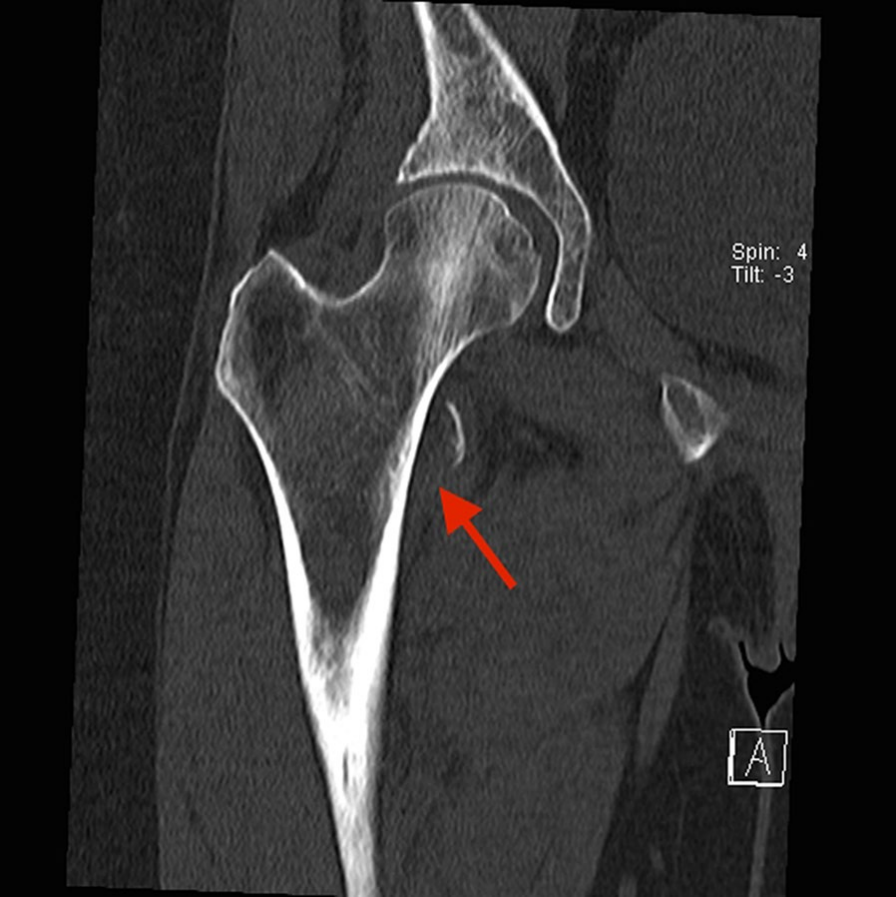
CT scan of the right hip and reconstruction in coronary view. The *red arrow* illustrates the avulsion fracture of the lesser trochanter

**Fig. 4:**
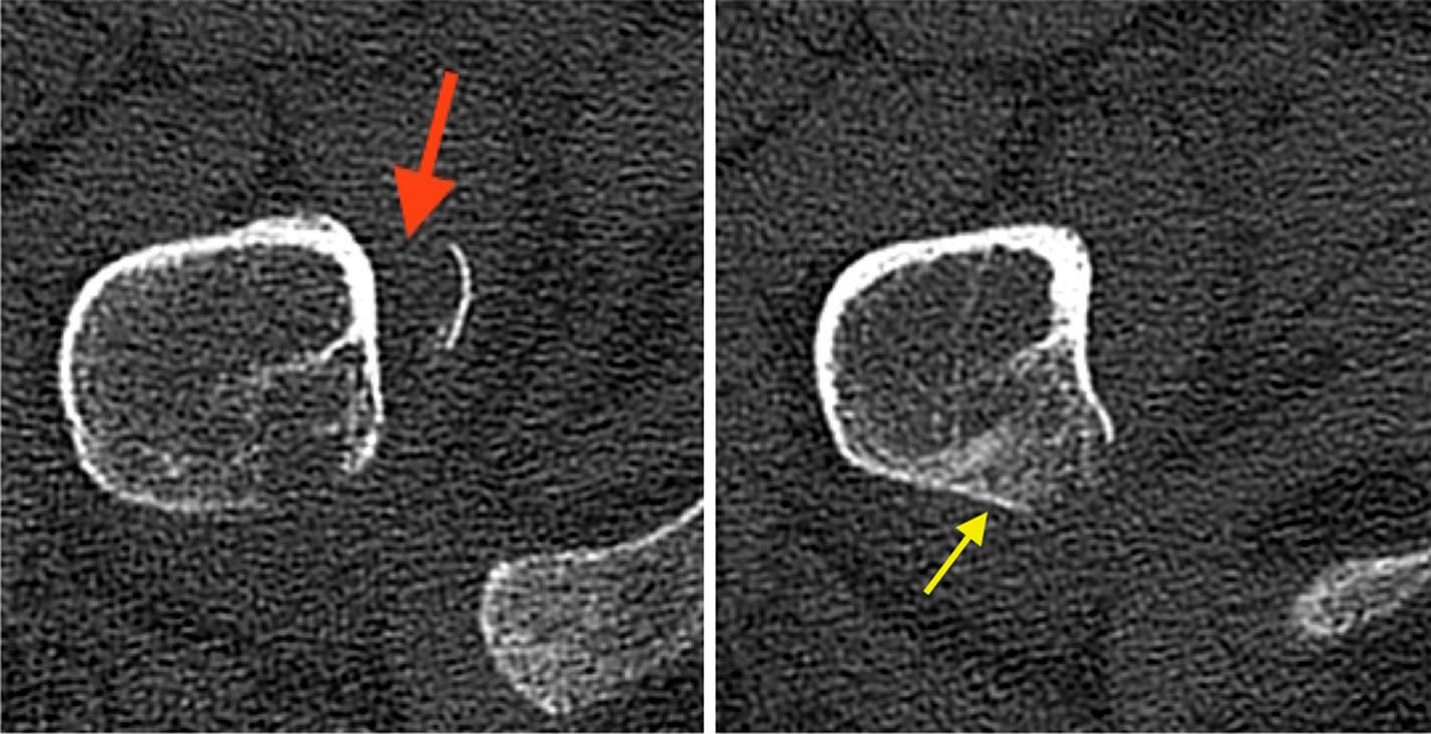
CT scan of the right trochanter area and reconstruction in axial view. Avulsion fracture of the lesser trochanter (*red arrow*) and majority of the lesser trochanter is intact (*yellow arrow*)

**Fig. 5:**
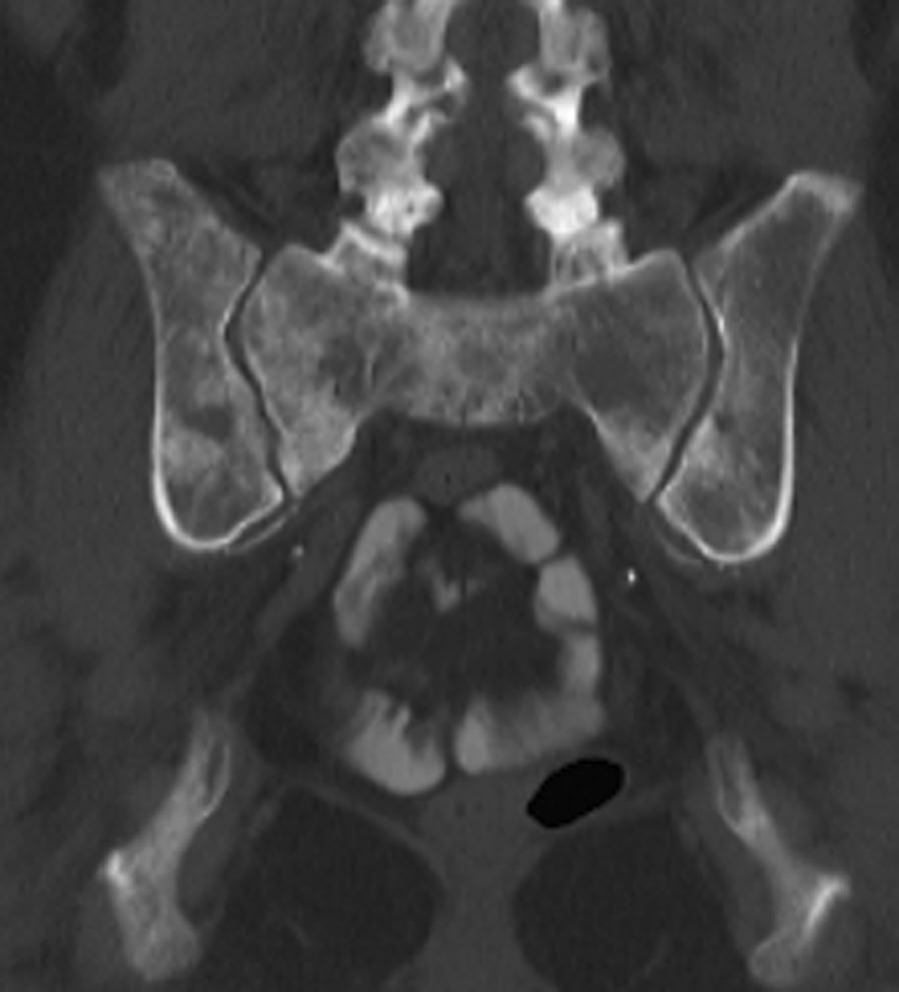
CT scan of the pelvis. Multiple osseous metastases of pelvis were detected

A 61-year-old woman, smoker (20 pack years), presented in the emergency department of our hospital with sudden onset of pain at the right groin after treatment by her chiropractor. Furthermore, she complained of moderate low back pain that was the reason for chiropractor treatment, started 4 months before. Furthermore, she described unexplained weight loss of 5 kg in 4 months. Sporadic onset of night sweats was also reported. She had no other musculoskeletal or constitutional diseases in her medical history. Physical examination showed tenderness in the right groin, almost preserved passive mobility of the right hip joint in the full range of motion. Psoas sign was positive during examination combined with limited active range of motion of the right hip joint. In addition, rising from a sitting position was extremely painful. Plain radiographs of the pelvis revealed an isolated fracture of the lesser trochanter with proximal displacement (see Figs. 1, 2). Furthermore, CT scans of the pelvis showed multiple osteolytic lesions in the pelvic ring (see Figs. 3, 4, 5).

On suspicion of a malignant process combined with a pathological fracture of the right lesser trochanter, hospitalization was decided for additional examination. Analgetics (metamizole, paracetamol) and anti-inflammatory treatment (ibuprofen) were prescribed with non-weight-bearing on two crutches. During hospitalization, extended clinical examination showed a palpable painless mass in the left breast with irregular edges. Sonograpically, a 1.75–1.14 cm round tumor in the upper inner quadrant of the left mamma was further specified. In mammography, no other tumor masses were detected. Being assessed in Breast Imaging Reporting and Data System (BI-RADS), the tumor was suggested as highly malignant. Further, punch biopsy was recommended. This showed a poorly differentiated carcinoma (high grade, G3); International B-Classification: B5b. The Her2-receptor status was negative. Progesterone and estrogen-receptor analysis resulted in an IRS score of 12 for progesterone, and 3 for estrogen.

Thoraco-abdomino-pelvic computerized tomography (CT) extension assessment revealed disseminated hepatic metastatic spread, multiple skeletal metastases in the pelvis, the thoracic and lumbar spine (1st, 2nd, 3rd thoracic vertebra and 2nd lumbar vertebra) and older pathological fractures of the 5th and 6th rib on the left and right thorax. No intrapulmonary process was detected. Finally, the tumor was classified as a poorly differentiated carcinoma (G3) of the left mamma (cT2, cN2, M1 (osseous and hepatic)). Blood tests showed a slight leukocytosis and moderate elevation of the C-reactive protein. Our hospital-internal interdisciplinary tumor committee discussed the case, and a palliative procedure was decided. The initiated palliative procedure consisted of chemotherapy with a weekly application of Paclitaxel and Bevacizumab. Re-evaluation of the lumbar spine and right hip joint has shown no fracture gap of the femoral cortex, except for the fracture of the lesser trochanter. Furthermore, no osteolytic processes have been shown. The trabecular structure of the femoral neck was preserved and the majority of the lesser trochanter complex was intact. According to known published recommendations [4], prophylactic stabilization of the right proximal femur with an intramedullary nailing system was discussed on the basis of possible instability. However, the patient refused surgical treatment at this time and decided upon conservative treatment. The patient was free of complaints and had no limitation in mobility after a follow-up period of 5 months.

## Discussion

Isolated fractures of the lesser trochanter in adults are uncommon [5, 6]. Most often it is seen as an avulsion in adolescent athletes [1]. Forceful traction and sudden distension by the iliopsoas tendon is thought to be the most common mechanism [2]. Commonly, affection of the lesser trochanter is seen in combination with complex fractures of the femoral neck or pertrochanteric area [7]. In the literature, a few cases of isolated lesser trochanteric fracture described a closed relation to an unknown tumor disease. First, in 1984, Bertin et al. presented a case series of 36 patients with an isolated fracture of the lesser trochanter. Of those, four cases were described as a result of secondary metastases (follicular adenocarcinoma of the thyroid, islet cell pancreatic carcinoma, prostate carcinoma and adenocarcinoma of unknown origin), direct trauma history was missing. Thus, the authors conclude that a thorough search for occult metastatic malignant diseases should be performed in case of atraumatic fractures in adults [8]. In 1998, Khoury et al. presented a case series of three patients. In one patient showing a medical history of infiltrative ductal of the right mamma, lesser trochanteric fracture was the first sign for metastatic disease. In another patient, there was no history of a primary tumor and the fracture was the first sign of disease [9]. In our case, avulsion of the lesser trochanter was the first osseous sign for metastatic disease as result of invasive cancer of the breast.

In 2006, James et al. noted in their case series of 15 patients that 69 % of these fractures are associated with metastatic diseases. Therefore further investigation should be made to detect an underlying bone infiltration [5]. Some authors recommend a magnetic resonance imaging (MRI) to estimate the extent of infiltration and bone stability [1, 5]. Afra et al. [10] and Heiney et al. [11] recommend a ^99^technecium scintigraphy to rule out other bone metastases. In our case neither MRI nor scintigraphy was performed, because thoraco-abdomino-pelvic CT scans revealed multiple metastases in the skeleton and showed a no fracture-threatened bone at the right hip joint.

Treatment of these patients depends on the stage of primary tumor. In view of the risk of impending femoral shaft fracture, prophylactic internal fixation with associated palliative treatment of the carcinoma is recommended in most cases [6, 8]. Haentjens et al. [4] showed in a review of the literature that prophylactic stabilization is needed in cases of metastatic involvement of the proximal femur combined with an isolated fracture of the lesser trochanter. In contrast to this, CT scans in our case revealed the known isolated fracture of the lesser trochanter, but further osseous metastatic infiltration of the trabecular structure was missing; especially the weight-bearing cortex of the proximal femur was intact. However, prophylactic stabilization can be discussed to reduce the risk for impending fracture of the proximal femur. The clinical findings have to be discussed with the patient and a case-by-case decision has to be made. In cases of a controlled primary tumor and single metastases, resection of the lesion and hip replacement is the most recommended treatment. According to a publication by Rouvillain et al. [3], complete metastasis-resection and implantation of a femoral megaprosthesis in a patient with metastatic lung adenocarcinoma in a stable state is an additional treatment possibility.

In general, osseous metastases originate in breast, lung or prostate tumors in most cases. The skeleton is the third most frequent site for carcinoma spreading after the liver and the lung. At this, common carcinomas are breast tumors in women and prostate cancers in men. In 25–30 % of these cases, skeletal lesions are the first sign of metastatic disease [12]. In case of breast cancer, bone is the first metastatic site in more than 50 % of patients [13]. The most common metastatic locations are the whole spine and the proximal femur [14]. There is an ongoing discussion about how to treat impending pathological fractures of the proximal femur. Surgery is the standard of treatment for enhancement of stability in those cases. Compared with surgical treatment in cases in that a pathological fracture has occurred, prophylactic surgical stabilization improves the patient’s quality of life with good outcome. Ward et al. [15] reported that the surgical treatment of impending femoral fractures yielded better results than the treatment of completed fracture, especially in cases of postoperative planned radiation. Haentjens et al. [4] recommend internal fixation in cases of pathological fractures that involve the lesser trochanter. The criteria outlines by Mirel et al. [16] should help the surgeon for the right treatment decision. Conservative therapy is another treatment possibility but little is known about treatment outcome. In summary, the right therapy depends on the patients’ status, the surgical intervention and the postoperative complications.

Known case reports describe a large variety of origin of malignancies. In our case, the lesser trochanteric fracture was the first evidence for metastatic spreading of a high-grade breast carcinoma. Further cases are needed for a detailed analysis of this type of fracture.

## Conclusion

Isolated avulsion of the lesser trochanter without adequate trauma is a rare presentation of hip fractures in adults. It is closely related to an unknown tumor disease. Therefore, we recommend further investigation to detect an underlying, usually metastatic tumor disease. Finally, treatment of these patients depends on the staging of the tumor disease.

## Consent

A written, informed consent was obtained from the patient for publication of this case report and any accompanying images. A copy of the written consent is available for review by the Editor-in-Chief of this journal.
